# Multiple paedomorphic lineages of soft-substrate burrowing invertebrates: parallels in the origin of *Xenocratena* and *Xenoturbella*

**DOI:** 10.1371/journal.pone.0227173

**Published:** 2020-01-15

**Authors:** Alexander Martynov, Kennet Lundin, Bernard Picton, Karin Fletcher, Klas Malmberg, Tatiana Korshunova

**Affiliations:** 1 Zoological Museum, Moscow State University, Moscow, Russia; 2 Gothenburg Natural History Museum, Gothenburg, Sweden; 3 Gothenburg Global Biodiversity Centre, Gothenburg, Sweden; 4 National Museums Northern Ireland, Holywood, Northern Ireland, United Kingdom; 5 Queen’s University, Belfast, Northern Ireland, United Kingdom; 6 Milltech Marine, Port Orchard, Washington, United States of America; 7 Gothenburg Global Biodiversity Centre, Aquatilis, Gothenburg, Sweden; 8 Koltzov Institute of Developmental Biology RAS, Moscow, Russia; Laboratoire de Biologie du Développement de Villefranche-sur-Mer, FRANCE

## Abstract

Paedomorphosis is an important evolutionary force. It has previously been suggested that a soft-substrate sediment-dwelling (infaunal) environment facilitates paedomorphic evolution in marine invertebrates. However, until recently this proposal was never rigorously tested with robust phylogeny and broad taxon selection. Here, for the first time, we present a molecular phylogeny for a majority of the 21 families of one of the largest nudibranch subgroups (Aeolidacea) and show that the externally highly simplified vermiform nudibranch family, Pseudovermidae, with clearly defined paedomorphic traits and inhabiting a soft-substrata environment, is a sister group to the complex nudibranch family, Cumanotidae. We also report the rediscovery of one of the most enigmatic nudibranchs–*Xenocratena suecica*–on the Swedish and Norwegian coasts 70 years after it was first found. *Xenocratena* was described from the same location and environment in the Swedish Gullmar fjord as one of the most enigmatic vermiform organisms, *Xenoturbella bocki*, which represents either an original simple bilaterian body plan or secondary simplification of a more complex organisation. Our results show that *Xenocratena suecica* reveals an onset of parallel paedomorphic evolution so we have proposed the new family, Xenocratenidae fam. n., to accommodate the molecular and morphological disparities we discovered. The paedomorphic origin of another aeolidacean family, Embletoniidae, is also demonstrated for the first time. Thus, by presenting three independent lineages from non-closely related aeolidacean families, Xenocratenidae fam. n., Cumanotidae and Embletoniidae, we confirm with phylogenetic data that a soft-substrata burrowing-related environment strongly favours paedomorphic evolution. We suggest criteria to distinguish ancestral and derived characters in the context of modifications of ontogenetic cycles. Applying an evolutionary model of the soft substrate-driven multiple paedomorphic origin of several families of nudibranch molluscs we propose that it is plausible to extend this model to other marine invertebrates and suggest that the ancestral organisation of the enigmatic metazoan, *Xenoturbella*, might correspond to the larval part of a complex ancestral bilaterian ontogenetic cycle with sedentary/semi-sedentary adult stages and planula-like larval stages.

## Introduction

Paedomorphosis is an important evolutionary force and has been identified in an array of animal groups including birds and humans [1,2]. Paedomorphic animals commonly demonstrate simplified features that correspond to early ontogenetic stages of an ancestral taxon [3,4]. However, assessments of infaunal-driven paedomorphosis in phylogenetic analyses are limited [5]. Previous proposals that a soft bottom marine environment (also called meiobenthic or interstitial) drives paedomorphic evolution in various invertebrates [6], in contrast with the suggestions that interstitial organisms appeared first in evolution [7], were never consistently tested. The importance of potential paedomorphosis-related evolution is illustrated by the most recent debates about the phylogenetic position of *Xenoturbella*, one of the most enigmatic but morphologically very simple vermiform animals only a few centimeters in length which burrows in a soft-substrate environment [8]. A relatively recent phylogenetic analysis placed *Xenoturbella* within the Deuterostomia [9], which implies evolution towards simplification from a more complex ancestor, but just a few years ago two studies placed *Xenoturbella* together with Acoelomorpha as a basal bilaterian offshoot and a sister-group to Nephrozoa (Protostomia and Deuterostomia) [10,11]. However, a recent phylogenetic study on tunicates also questioned this basal position of *Xenoturbella* [12], which again raised the question about the organisation of the xenacoelomorphan ancestors.

**Fig 1:**
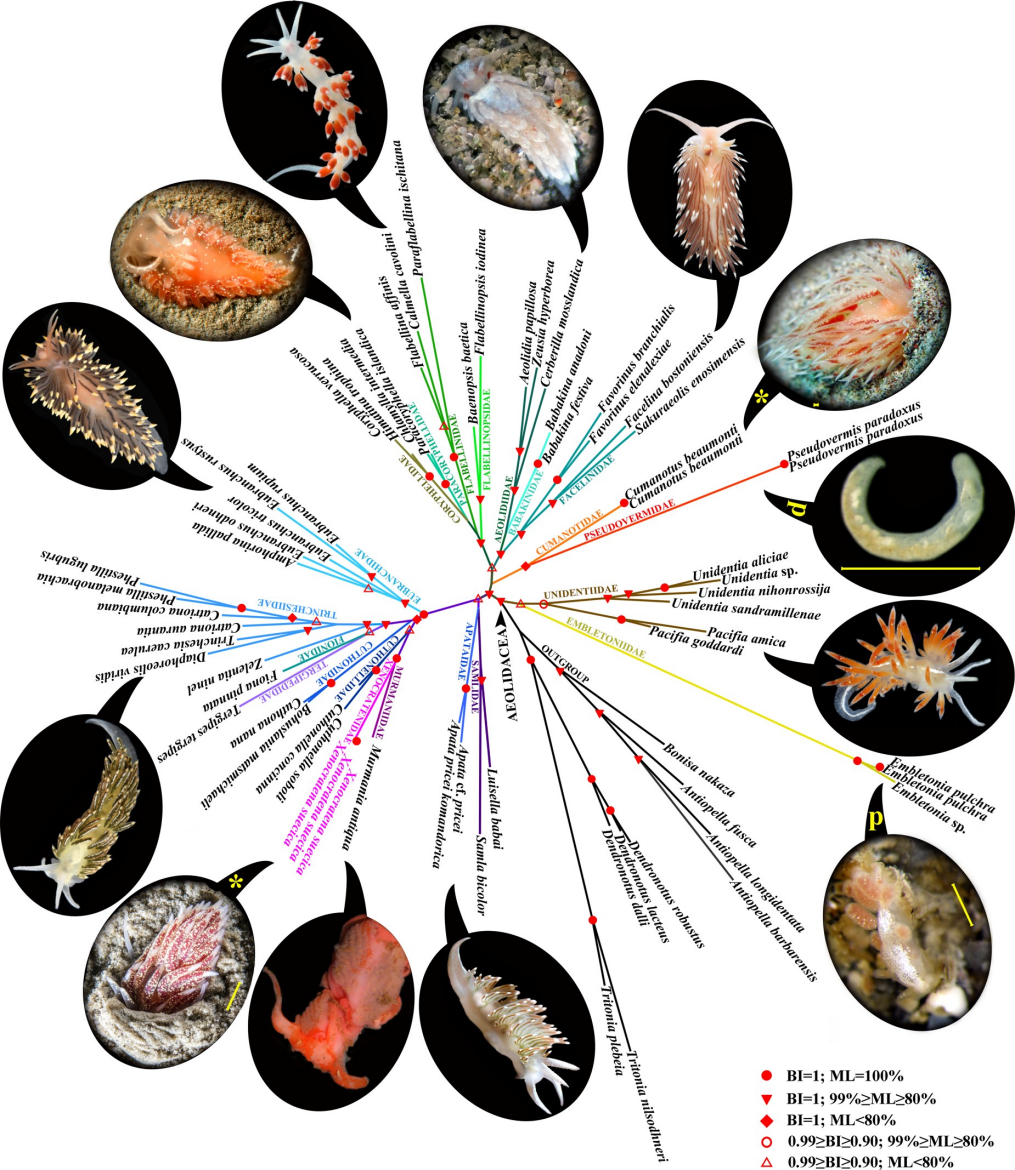
Phylogenetic tree of aeolidacean nudibranchs based on concatenated molecular data (COI + 16S + 18S + 28S + H3) represented by Bayesian inference (BI). Posterior probabilities from BI and bootstrap values for Maximum Likelihood (ML) indicated on the figure. Highly simplified paedomorphic families Pseudovermidae and Embletoniidae, indicated by the letter P. Two distantly related families Xenocratenidae and Cumanotidae showing maximal convergence in external shape and ability to facultively burrow in soft substrate indicated by yellow asterisks. Scale bars 1 mm.

**Fig 2:**
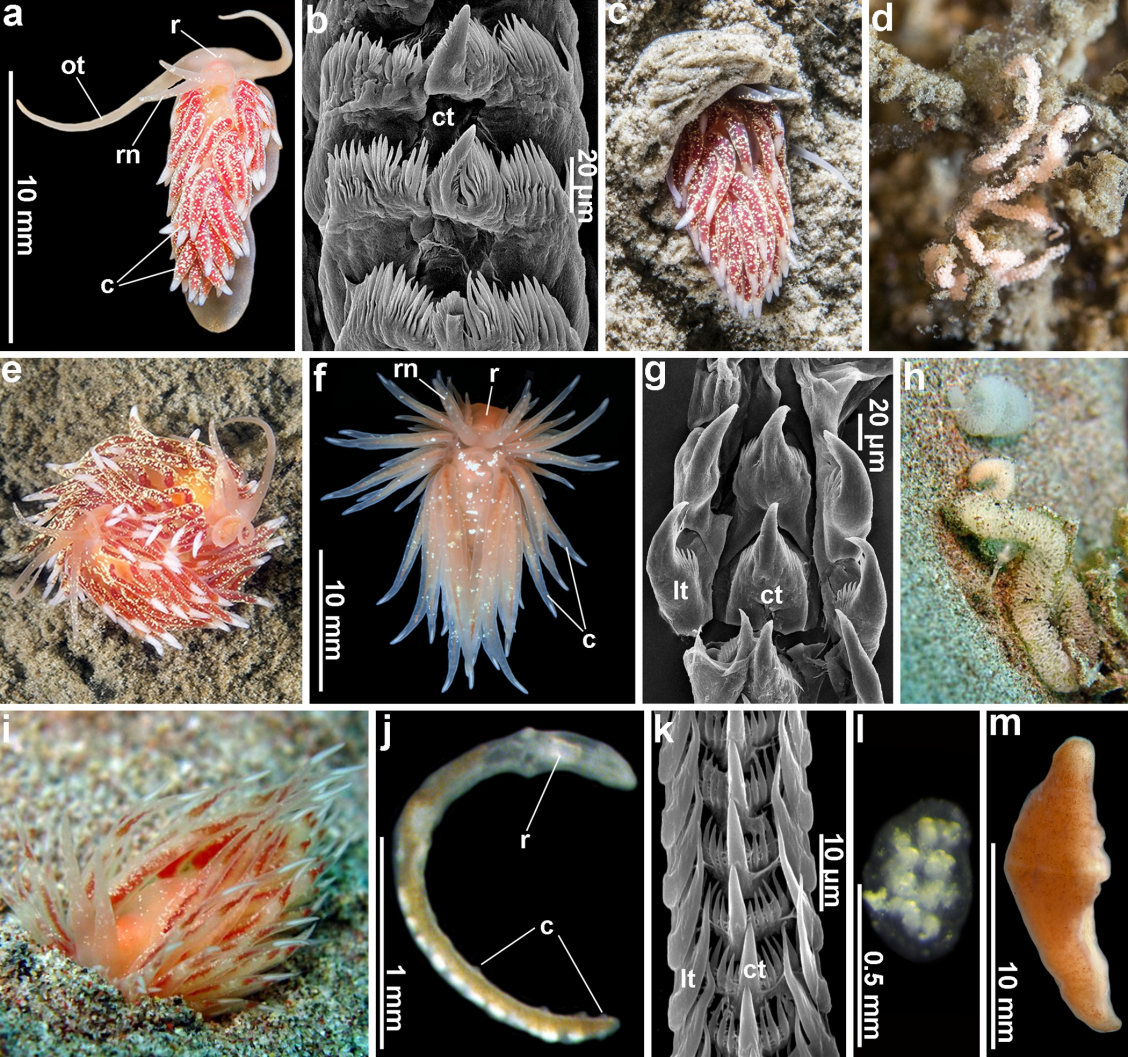
Nudibranch taxa *Xenocratena suecica* (A–E) and *Cumanotus beaumonti* (F–I) with facultative ability to burrow into soft substrata (C, I) compared to the highly simplified obligate infaunal *Pseudovermis paradoxus* (J–L) and *Xenoturbella* (M). Note the high external and behavioral similarity between the phylogenetically distantly related *Xenocratena* (A, C, E) and *Cumanotus* (F, I) and the drastic external differences between the phylogenetically closely related complex *Cumanotus* (F, I) and paedomorphic vermiform *Pseudovermis* (J). Radula (molluscan teeth) features, on the contrary, are very different between *Xenocratena* (B, note the absence of lateral teeth, lt) and *Cumanotus* (G, note the presence of lateral teeth, lt) and similar between *Cumanotus* (G) and *Pseudovermis* (K, note the presence of lateral teeth, lt). Both *Xenocratena* (D) and *Cumanotus* (H) are able to lay egg masses facultatively on soft substrata, whereas *Pseudovermis* (I) lays egg masses obligately on the soft substrate. Abbreviations: c, cerata (dorsal papillae); ct, central teeth of radula; lt, lateral teeth of radula; ot, oral tentacles; r, area below which radula is placed; rn, rhinophores (chemical sense organs).

**Fig 3:**
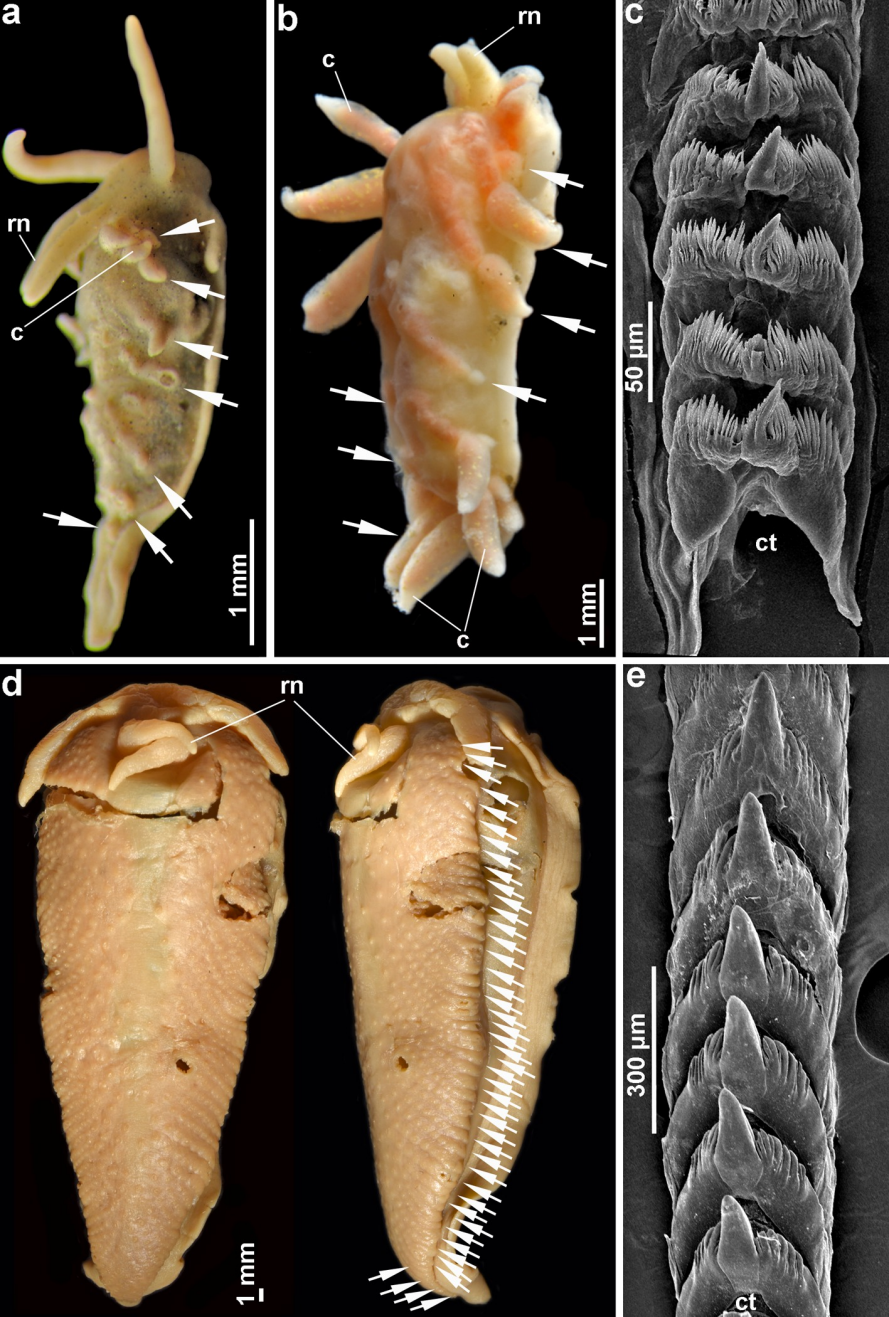
Comparison of phylogentically sister, complex non-burrowing *Murmania antiqua* (Martynov, 2006) (family Murmaniidae) from the Arctic Ocean with simplified facultative burrowing *Xenocratena suecica* Odhner, 1940 (family Xenocratenidae fam. n.) from the Atlantic (North Sea) waters of Sweden and Norway. The degree of simplification of ceratal (papillae-bearing) rows (every row is indicated by a separate arrow, majority of papillae were removed to clearly show the number of ceratal rows, which are counted according to the knob-like traces of the ceratal attachment on the dorsal side) can be evaluated directly between *Xenocratena* (A, B) and *Murmania* (D). *Xenocratena* (A, B) possesses no more than five (commonly fewer) of anterior ceratal rows and no more than eight (commonly fewer) ceratal rows in total. *Murmania* (D) possesses more than 20 anterior ceratal rows and more than 40 ceratal rows in total. Both small syntype (A, preserved specimen 4 mm in length) and newly discovered specimens (B, preserved, length 7 mm) specimens of *Xenocratena suecica* are compared with the large holotype specimen of *Murmania antiqua* (D, preserved, length 44 mm). The radular teeth of *Xenocratena* (C) and *Murmania* (E) are also presented to show the considerable morphological differences between these phylogenetically sister taxa. Abbreviations: c, cerata (dorsal papillae); ct, central teeth of radula; rn, rhinophores (chemical sense organs).

Because recent molecular phylogenetic data on *Xenoturbella* is still contradictory, we propose the application of a model of its evolution parallel to other organisms from the same environment. A potentially suitable model should include a robust phylogeny inferred for a large phylogenetically well-investigated predominantly complex group, but also one that contains a few examples of evidently derived simplified paedomorphic taxa which emerged within fundamentally the same soft-substrata environment as *Xenoturbella*. Here we suggest using the large and diverse group of nudibranch molluscs as such a model. Nudibranchs are a fascinating group of shell-less molluscs that demonstrate astonishing diversity and peculiar evolutionary traits [13,14] and are commonly used as model organisms in neurophysiology and other fields [15,16]. A subgroup of nudibranchs, treated here as suborder Aeolidacea, has evolved a defensive system for the secondary usage of cnidocysts from cnidarian prey [17, 18]. While the phylogeny of Aeolidacea is currently actively studied [18–21], a majority of these nudibranchs as defined within the previously established broad-scope aeolidacean nudibranch phylogenetic framework [19], are complex animals with numerous dorsal papillae (cerata) (Figs 1–3). However, there is a single exclusively infaunal family, Pseudovermidae, with evident paedomorphic features such as vermiform shape and reduced dorsal papillae, which strongly matches early ontogenetic stages of complex aeolidaceans [22]. This unique aeolidacean nudibranch family has never been assessed with molecular phylogenetic methods prior to this study.

Therefore, inclusion of the profoundly paedomorphic family, Pseudovermidae, into a robust phylogeny with a majority of other complex aeolidacean families will lead to the development of the above outlined model of evolution which can be further applied to the study of evolution of other meiobenthic and soft-substrata infaunal groups with uncertain phylogenetic position, like *Xenoturbella* [8,10]. Remarkably, in about the same time period as *Xenoturbella* was described (the 1940s), a nudibranch mollusc was described with a quite similar name–*Xenocratena suecica* [23]–from the same body of water with low circulation (Gullmar fjord, Sweden), the same bottom environment (soft mud) and at a similar depth (30–45 m), as the widely known *Xenoturbella bocki* [24]. However, unlike *Xenoturbella*, the nudibranch *Xenocratena* was never found again prior to this study and was never before involved in any molecular phylogeny. Thus, *Xenocratena*, a medium-sized (10–12 mm) animal from a soft-bottom environment, together with the smaller (up to 6 mm) paedomorphic infaunal family, Pseudovermidae, and other soft-bottom dwelling nudibranchs, are relevant to the present study in the assessment of a primary or secondary origin of regressive organisation.

## Materials and methods

### Sample data

Material for this study was obtained from various fieldworks, and from the following museums, the National Museums Northern Ireland, Cultra, Belfast, Gothenburg Natural History Museum (GNM), Norwegian University of Science and Technology, Swedish Museum of Natural History (Stockholm), Zoological Museum of Lomonosov Moscow State University (ZMMU), and other institutions. Specimens of *Xenocratena suecica* were observed and collected alive by SCUBA diving in 2017 and 2018 in southwestern Sweden and Norway, Idefjord, Sweden (less than 100 km from the type localities of *Xenocratena suecica* and *Xenoturbella bocki* in the Gullmar Fjord, at 59°5´ N, 11°17´ E, June 2017, 25 m depth, collected by Michael Lundin), and in Døvik, near the city of Stavanger, in the Rogaland region on the Norwegian south west coast (ca. 300 km from the type locality of *X*. *suecica* in Sweden, at 59°7´ N, 6°5´ E, 22 m depth, 5 May 2018, 12 June 2018, collected by Rudolf Svensen and Leif Bruntveit) (Fig 2A–2E). The bottom substrate at the Norwegian locality is soft and fine, silty muddy sand with the sea pen *Virgularia mirabilis* (Müller, 1776) and the hydroid *Corymorpha nutans* M. Sars, 1835, both potential food objects of *X*. *suecica*. Other nudibranchs seen at the locality are *Armina loveni* and *Cumanotus beaumonti* (Eliot, 1906) (family Cumanotidae, see Discussion). In total more than 30 specimens of *X*. *suecica* were observed in situ alive. Thirteen specimens were preserved for molecular and morphological study and deposited in the Gothenburg Natural History Museum. No specimens were observed feeding, suggesting that feeding could occur when burrowing. When disturbed, the nudibranchs quickly delve down into the bottom substrate (Fig 2C) and disappear completely, thus demonstrating evident facultative burrowing behaviour. Other verified observations of *X*. *suecica* were made in Egersund, Norway, May 2017, by Erling Svensen (without collecting). *Xenoturbella bocki* was collected by Kennet Lundin during a survey in the Gullmar fjord (Sweden) in April of 2016 at depths of 30–40 m. In addition, the original type specimens of *X*. *suecica* in the Swedish Museum of Natural History (Stockholm) and the Gothenburg Natural History Museum, collected in Gullmar fjord, Sweden were investigated (Fig 3A). Three samples with type material collected at 25 m, 35 m and 35–40 m depths were available for study. The length of newly discovered living specimens observed in this study was from 10 to 12 mm. No permission was necessary to obtain samples in the field and to access the museum collections. The morphology of the nudibranchs and their egg masses were studied under a stereomicroscope and using Nikon D810 and Nikon D600 digital cameras. For the description of internal features, both preserved and fresh specimens (when available) were dissected under the stereomicroscope. The buccal mass of each specimen was extracted and processed in 10% sodium hypochlorite solution. The coated radulae were examined and photographed using a scanning electron microscope (CamScan, JSM).

### Molecular analysis

Small pieces of tissue were used for DNA extraction with Diatom™ DNA Prep 100 kit by Isogene Lab and the protocol provided by the manufacturer. A commonly used set of markers were sequenced: mitochondrial cytochrome c oxidase subunit I (COI) and 16S rDNA, and nuclear Histone 3 (H3), 18S rDNA and 28S rDNA. The primers and polymerase chain reaction programs used are presented in the supporting information, S1 Table. Protein coding sequences were translated into amino acids for confirmation of the alignment. All newly generated sequences were deposited in GenBank (Supporting information, S2 Table, highlighted in bold). All new and publicly available sequences were checked via BLAST searches in GenBank (https://blast.ncbi.nlm.nih.gov/Blast.cgi) to verify identification and against potential contaminations. Original data and publicly available sequences were aligned with the MAFFT algorithm [25]. Separate analyses were conducted for COI (657 bp), 16S (488 bp), H3 (327 bp), 28S (336 bp), 18S (1808 bp) and five concatenated markers (3616 bp). Gblocks 0.91b [26] was applied to discard poorly aligned regions for the 18S data set (using less stringent options; 20% of the positions were eliminated). The GTR + I + G model was chosen for the concatenated dataset using MrModelTest 2.3 [27] under the Akaike information criterion [28]. Two different phylogenetic methods, Bayesian inference (BI) and Maximum Likelihood (ML), were used to infer evolutionary relationships. Bayesian estimation of posterior probability was performed in MrBayes 3.2 [29]. Four Markov chains were sampled at intervals of 500 generations. Analysis was started with random starting trees and 10^7^ generations. ML analysis was performed using RAxML 7.2.8 [30] with 1000 bootstrap replicates. Final phylogenetic tree images were rendered in FigTree 1.4.2. Ancestral character state reconstruction for paedomorphic traits were run using the maximum likelihood model Mk1 in Mesquite v3.10 [31], based on the topology of the best tree from the RAxML analysis of a concatenated dataset.

### Criteria for distinguishing the paedomorphic state in metazoans

Distinguishing secondary paedomorphic-related simplification from an initial simple ancestral body plan is a difficult evolutionary challenge. A commonly applied definition of paedomorphosis (including “progenesis” and “neoteny”) noted in the literature as a “retention of ancestral juvenile characters in the descendant adult phase…” [32] does not imply a method for phylogenetic polarization [33]. Therefore, an original ancestral adult state potentially can be misidentified with a derived larval state in a paedomorphic descendant, and vice versa. Two following operational criteria are used here to distinguish between primary earlier developmental characters of an ancestor and the secondary appearance of early developmental features at the adult state of a paedomorphic descendant: 1) A set of larval or early postlarval features common for many non-closely related taxa of a large taxonomic group, which appear at the adult stage of a paedomorphic group in question with a combination of specialized/unique adult characters that is restricted only to some particular group of related taxa of the higher-level taxonomic group; 2) Molecular phylogenetic data that place a potential paedomorphic group inside of a larger group with otherwise non-paedomorphic adult morphology. Previously, the successful application of these two criteria to distinguish between an ancestral early developmental state and a derived paedomorphic state has been shown, using two very disparate metazoan phyla, molluscs and echinoderms. Particularly, for nudibranch molluscs of the family Corambidae the ancestral status has been indicated for a long time. However, it was shown that corambids display external characters that are common in early postlarval stages of various and not directly related families of a higher level group, dorids, to which Corambidae belong, and at the same time corambids possess specific characters of the adult buccal apparatus that are present only in few related dorid families, but not in any group outside of the dorids [34–37]. Such morphological data thus fulfill the first criterion of evidence for secondary paedomorphic organisation. The morphological criterion well agrees with recent molecular data in which the Corambidae never appear as basal-most dorids but are placed inside of the group of families which possess exactly the same type of specialized buccal apparatus [36, 38]. A parallel case is represented by ophiuroid echinoderms, where previously some strongly simplified groups were considered ancestral ones [39], however it was shown that such groups have specific features that are highly similar to the postlarval stages of common ophiuroid families with complex adult stages [40, 41], thus fulfilling the first criterion of paedomorphosis. Most recently, a genome-scale analysis of a whole class of Ophiuroidea confirmed that previously assessed”ancestrally simple” ophiuroid taxa are nested within ophiuroid families with complex adult morphology [42]. Thus, these examples of distinguishing early developmental ancestral stages and derived paedomorphic states from two very different metazoan phyla demonstrate the practical usefulness of these two major criteria to detect secondary paedomorphic/ontogenetically altered states. With some care, these criteria can be applied to other metazoan groups.

An additional third criterion for potential evaluation of paedomorphic organisation is ecological criterion. This additional criterion should be applied especially carefully, because it is indirect, compared to the two main criteria. For example, it is well established that deep-sea environments facilitate the appearance of paedomorphic organisms, including such different groups as fishes [43] and ophiuroids [40,41]. An environment may contain non-paedomorphic groups as well, so such ecological information cannot be used solely as an indicative criterion and must be checked against the two main ones. There is evidence that brackish water environments also facilitate developmental retardations, resulting in paedomorphosis. This is particularly well exemplified by the recent discovery of a peculiar new brackish water nudibranch genus and species [44], in a Swedish fjord. Finally, the soft-bottom meiobenthic environment has been considered a strong driver of paedomorphic organisation [6, 22], thus being a very reliable example of ecological criterion for paedomorphosis. However, it needs further verification with morphological data and molecular phylogeny on different metazoan groups.

For practical applications it is also important to clarify the definition of the term paedomorphosis and the related conceptions of “progenesis” and “neoteny”. The term paedomorphosis was suggested in a broader sense by Garstang in 1928 [45] to encompass various phenomena of the appearance of larval characters of ancestors at the adult stages of descendants and to highlight its role in macroevolution, specifically in deuterostomian evolution [46], thus initially giving that term a broad definition and evolutionary and phylogenetic application. The related terms “progenesis” and “neoteny” were instead originally proposed for very restricted cases in particular taxonomic groups without phylogenetic context. “Neoteny” was proposed by Kollman in 1885 [47: 391] specifically to describe the retardation of development in some amphibian species (including axolotl), and though retardations in yeasts and plants were also mentioned, he clearly described “neoteny" as an intraspecific process, not an interspecific, phylogenetic one as is now commonly attributed to human evolution in particular. “Progenesis” was first suggested by Giard in 1887 in reference to precocious maturation in some decapod crustaceans due to parasitic castration, without a direct link to evolutionary processes [48: 23]. Giard [48] also referred to axolotl larvae, among other cases, thus his “progenesis” term echoed the definition of Kollman’s earlier “neoteny” term [48] with both terms clearly focused on individual adaptations and intraspecific processes, not phylogenetic ones.

The term “paedomorphosis” is currently universally accepted as a higher-level term encompassing both “neoteny” and “progenesis”, however, the latter term especially is sometimes used separately and as a substitute for the term paedomorphosis [49]. This is incorrect because, as was shown above (see also [45]), originally the terms “progenesis” and “neoteny” lacked the key phylogenetic component and were highly inconsistent with the initial and modern meanings of the term paedomorphosis. The traditionally used definitions of “progenesis” (abrupt cessation of ontogenetic development, leading to appearing smaller compared to ancestors of mature organisms with strongly expressed larval features [32], with evident examples in several polychaete lineages [50]) and “neoteny” (slow development of some set of characters, resulting in appearing larger compared to ancestor organisms with a commonly resulting mixture of underdeveloped and more complex characters, as for example in humans [2, 51, 52, 53] were controversially applied much later. Another crucial consideration is that “progenetic” and “neotenic” patterns are just different sides of the same paedomorphic process [32, 54]. In various organismal groups, often a taxon that demonstrates evident juvenile characters at the adult state is difficult to attribute exactly to “progenetic” or “neotenic” ones due to a strong heterochronic mosaicism of delayed and accelerated growth characters [41, 52, 53, 55, 56]. Contrasted paedomorphic pathways can also occur within same species [57] and there are many cases when a species demonstrates only partial paedomorphosis in some particular characters [4]. Thus, the original definitions of “progenesis” and “neoteny” did not refer to the evolutionary heterochronic processes, *per se*, and did not necessarily link shifting maturation time with somatic differentiations, therefore, ‘hypomorphosis’ was previously suggested as a substitute for the term “progenesis” and ‘deceleration’ substituted for the term “neoteny” [58]. “Neoteny”, however, is not an interchangeable synonym of paedomorphosis since the latter term was initially directly linked to phylogeny [45], whereas “neoteny” was not [47]. The term paedomorphosis includes both “progenesis” and “neoteny”, as well as more rarely mentioned processes, such as post-displacement [32]. Therefore, the general term paedomoprhosis is preferable for current usage and more importantly avoids the pitfalls of the numerous inconsistent definitions of “progenesis” and “neoteny” [55, 58]. While in biology at a general scale the importance of paedomorphosis has been recognised [51, 59], the fields of systematics and phylogeny (both at theoretical and practical levels) essentially remains ontogeny-free, and only recently was a special field of research of *ontogenetic systematics* [4, 36, 40, 41, 60, 61] outlined that targets linking such fundamental, but still very loosely connected, disciplines as evo-devo and taxonomy.

Paedomorphosis, in turn, is a part of broader ontogenetic processes, heterochronies (different timing of character appearance in ontogeny), which are responsible for both reduction and appearance of more complex characters in evolution [46, 54, 56–59, 62–68]. Though heterochronic retardations of character development in the course of general shifts in life cycles evolution are a major factor for both evolutionary reductions and novelties [60, 69], not all reductions can be directly considered paedomorphic ones. During the preceding phylogenetic history of a taxon, the reduction of a character can have occurred that may have led to a progressive evolution. For example, gill reduction became an important ground for the appearance of the prolific and very successful terrestrial vertebrate clades. The secondary reappearance of juvenile aquatic gills at the adult state of some amphibian clades is thus a strong paedomorphic event, whereas loss of such gills at the adult state in a majority of amphibians is not directly linked to paedomorphosis, though it is indeed part of broader ontogenetic heterochronic processes [33, 64, 70].

**Fig 4:**
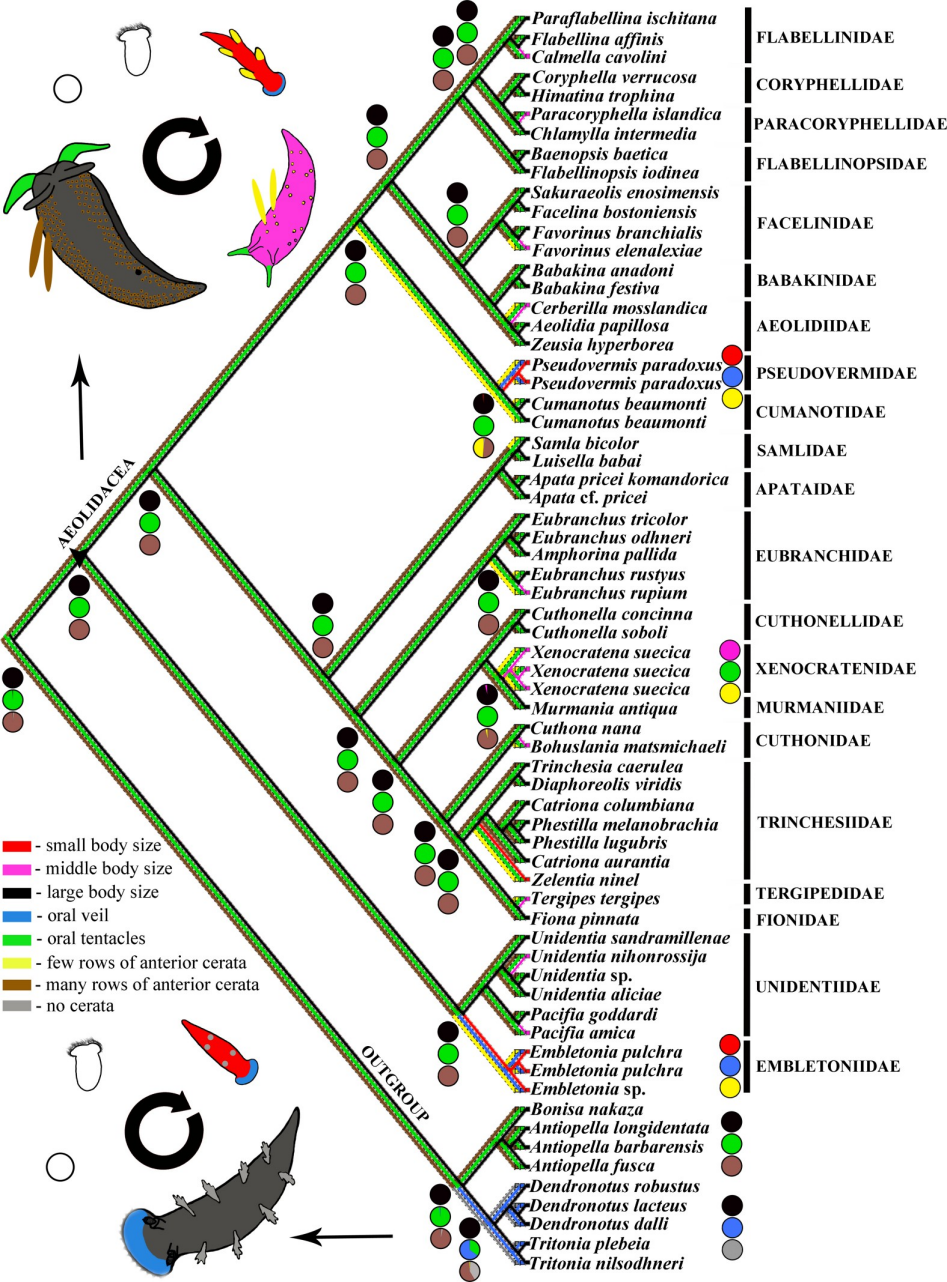
Ancestral state reconstruction for paedomorphic traits based on ML analysis of a concatenated dataset. Eight morphological characters are color-coded for analysis and additionally patterned on developmental stages of ontogenetic cycles. See details in the text. Pie charts represent degree of support for each character state at the nodes. Strongly paedomorphic adults characterized by small size (red), small oral veil (blue) and oral tentacles absent, and small number of anterior ceratal rows (yellow).

At a large scale, the differences between the acquisition of novelties, the reduction of characters and paedomorphic traits are not absolutely defined, and to some degree they overlap. To make these terms more precise, here we use several operational criteria to distinguish paedomorphosis in a narrow sense from heterochronic reductions in nudibranch molluscs. For example, currently it is universally accepted that the last common ancestor of nudibranch molluscs possesses a large broad oral veil without definite oral tentacles, similar to the modern Pleurobranchida, or dendronotacean nudibranchs, Tritoniidae [71–73, an outgroup, Fig 4]. Such an oral veil was further reduced or transformed in various nudibranch clades. The last common ancestor of aeolidacean nudibranchs, in turn, is characterized by a reduced oral veil and acquisition of solid oral tentacles [71]. The loss of a primary oral veil, though indeed a heterochronic event, was not immediately a paedomorphic one since it was connected with the acquisition of an important, progressive aeolidacean nudibranch novelty–oral tentacles. Correspondingly, at the phylotypic stage/period [36] (previously termed “recapitulation”) in the early ontogeny of all aeolidaceans the ancestral small oral veil appeared for a short time, then towards the adult state it was substituted with oral tentacles (Fig 4). However, inside of the large aeolidacean clade (Figs 1 and 4) there are a few cases where a small oral veil is strongly similar to the postlarval transient oral veil, but not to the elaborate ancestral veil of non-aeolidacean nudibranchs (Fig 4). Therefore, the small oral veil which occurrs in a few aeolidacean members, evidently secondarily (and with a clear correspondence to early postlarval features), is a strong sign of a paedomorphic trait. Further, aeolidaceans acquired another key evolutionary novelty–numerous outgrowths of the dorsal side (termed cerata), which are placed in respective rows or clusters. The cerata are a complex structure which appeared already in the last common ancestor of all aeolidaceans [18]. According to both morphological and molecular data [18, 71], the family Janolidae, which are comprised of large animals, commonly more than 20–30 mm in length with short oral tentacles and numerous ceratal branches (more than 10 ceratal branches in the adult state) without a clear distinction of the anterior and posterior rows (Fig 4, the janolid genera *Antiopella* and *Bonisa* as an outgroup), is basal to aeolidacean nudibranchs. Within aeolidaceans non-paedomorphic representatives attain a size of more than 15–20 mm, possess well-defined, long oral tentacles, and the differentiation of anterior and posterior ceratal branches in many families has occurred.

During ontogeny of all aeolidacean nudibranchs, a few juvenile ceratal rows (1–2 anterior rows in earlier postlarval stages, about 3–4 anterior rows in more advanced juveniles) precede the adult state with numerous ceratal rows (commonly more than ten in total, more than four in anterior rows) [22, 71]. Therefore, the presence of a smaller number of ceratal rows in the adult stage is a sign of at least partial paedomorphosis, and not just an overall reduction/loss, especially if such small-sized taxa with a smaller number of anterior ceratal rows are sister to large-sized taxa with numerous cerata. The following main characters are used here to detect paedomorphic states in aeolidacean nudibranchs: 1) Presence of a small oral veil instead of defining oral tentacles; 2) Small number of ceratal rows (less than four in anterior rows); 3) Smaller size (less than 10 mm in adult state; at this size juveniles commonly continue to increase the number of ceratal rows). Decelerated (“neotenic”) forms, in turn, may display clear juvenile features but at the same time be similar in size or larger than complex non-paedomorphic ancestors [32, 58]; we expect such processes within aeolidacean nudibranchs, too. The various combinations of such characters lead us to propose at least two main degrees of paedomorphosis in aeolidacean nudibranchs: A) Strongly (almost completely) paedomorphic adults (small oral veil (oral tentacles absent), small number of anterior ceratal rows, commonly small size); B) Moderately (“partially”) paedomorphic adults (oral tentacles present, small number of ceratal rows, small size) (Fig 4).

### Nomenclatural acts

The electronic edition of this article conforms to the requirements of the amended International Code of Zoological Nomenclature, and hence the new names contained herein are available under that Code from the electronic edition of this article. This published work and the nomenclatural acts it contains have been registered in ZooBank, the online registration system for the ICZN. The ZooBank LSIDs (Life Science Identifiers) can be resolved and the associated information viewed through any standard web browser by appending the LSID to the prefix "http://zoobank.org/". The LSID for this publication is: urn:lsid:zoobank.org:pub: 9DD489FE-58F2-44E4-B3CE-3AA74A0D10DF. The electronic edition of this work was published in a journal with an ISSN and has been archived and is available from the following digital repositories: PubMed Central, LOCKSS.

## Results

### Molecular phylogeny confirms the paedomorphic origin of highly simplified vermiform nudibranchs

Here, for the first time, we include five genes (the mitochondrial genes cytochrome oxidase subunit I (COI) and 16S rRNA, and the nuclear genes Histone 3 (H3), 18S rRNA and 28S rRNA) in the phylogenetic analysis (Fig 1) of representatives of a majority of the families of the Aeolidacea with the inclusion of the enigmatic genus, *Xenocratena* (Fig 2A, 2C and 2E), soft bottom-dwelling Cumanotidae [74] (Fig 2F and 2I), infaunal Pseudovermidae [75] and Embletoniidae [17,18] (Fig 2J). Bayesian Inference (BI) and Maximum Likelihood (ML) analyses based on the combined dataset yielded trees with identical topologies (Fig 1). Surprisingly, *Xenocratena* was placed in a sister clade (PP = 1, BS = 100%) with the morphologically very different complex family Murmaniidae from the Arctic [19, 76] (Figs 1–4), and not with the externally highly similar family, Cumanotidae (Figs 1 and 2F and 2I), from the same localities.

Like *Xenocratena* (Fig 2C), Cumanotidae facultatively burrow in soft bottoms (Fig 2I) and show similar external traits (Fig 1, marked by yellow asterisks), however according to the present molecular phylogeny Cumanotidae, even more unexpectedly, are sister to the family Pseudovermidae (Fig 1, marked by “p”) and not related to *Xenocratena*. Thus, our robust phylogeny for the first time shows that Pseudovermidae is evidently sister to a complex aeolidacean of the family Cumanotidae and placed inside of the generally complex Aeolidacea (Fig 1). According to the present morphological analysis, Pseudovermidae also possess a triserial radula similar to the sister Cumanotidae (Fig 2). Thus, two of the main criteria of paedomorphic secondary organisation outlined above are satisfied by the combined morphological and molecular evidence. The additional ecological criterion is also verified since strongly paedomorphic Pseudovermidae are exclusively meiobenthic organisms burrowing within soft substrata.

Confirmation of the paedomorphic origin of the family Pseudovermidae is also important because this is one of the most enigmatic and modified forms of all in the molluscan phylum (comparable to the profound modifications in aplacophoran molluscs [77]). According to the present evidence, it is plausible to infer that the common ancestor of cumanotids and pseudovermids may represent early stages of paedomorphosis-related soft bottom-dwelling adaptations, which would have further led to a reduction of the dorsal papillae and the worm-like body shape of the pseudovermids (Figs 1, 2 and 4). It has previously been documented that at early ontogenetic stages, non-burrowing aeolidacean nudibranchs could facultatively occur within the soft-substrata environment [22], and this might have facilitated a further evolutionary shift towards obligate infaunal habitats. Both *Xenocratena* and *Cumanotus* occasionally deposit egg masses directly on soft substrata (Fig 2D and 2H), thus favouring a potential evolutionary shift to infaunal habitats. Paedomorphic *Pseudovermis* places small egg masses obligately on a soft substrate (Fig 2L).

### Burrow-driven evolution results in multiple paedomorphic lineages of nudibranchs

We reveal notable convergence in the onset of burrow-driven evolution toward simplification within adult stages of several non-related taxa of Aeolidacea, including the rediscovered *Xenocratena* and the family Cumanotidae (Figs 1 and 4). This was a reason why, prior to this study, *Xenocratena* was formerly associated with distantly related aeolidacean groups [78]. The facultatively burrowing *Xenocratena* (Fig 2C) readily differs from the phylogenetically related Murmaniidae which, compared to *Xenocratena*, has a significantly larger size and more numerous rows of dorsal papillae (cerata). The degree of paedomorphic simplification of ceratal rows of *Xenocratena*, compared to *Murmania*, can be measured directly (Fig 3A, 3B and 3D). *Xenocratena* has no more than eight ceratal rows (Fig 3A and 3B, indicated by arrows), whereas *Murmania* possesses more than 40 (Fig 3D). Because during ontogeny of nudibranchs the number of ceratal rows gradually increases [79, 80], and because the common ancestor of all aeolidacean possessed a large number of ceratal rows [19] it then follows that the small number of ceratal rows in *Xenocratena* only corresponds to juvenile stages of a common ancestor of the clade (Figs 1 and 4) consisting of the small *Xenocratena* (commonly less than 10 mm, maximum 12 mm adult length) and large Murmaniidae (up to 46 mm adult length). Basal members of the unrelated aeolidacean families Aeolidiidae and Paracoryphellidae are also large-sized (adults up to 50 mm or more) with numerous ceratal rows and demonstrate considerable external similarities to the complex Murmaniidae [19]. This is evidence that such organisation is plesiomorphic in aeolidaceans and also fulfills the first (morphological) criterion for assessing paedomorphosis.

The facultative-burrowing adult *Xenocratena*, therefore, demonstrates early stages of paedomorphic evolution, whereas obligate infaunal Pseudovermidae instead demonstrate its ‘terminal stages’, when adult animals closely resemble early postlarval stages of other aeolidacean nudibranchs [22, 80]. Well-established examples from groups of vertebrates show that an irreversible fixation of originally facultative/intraspecific paedomorphic traits [58] is part of the evolutionary process [81]. Additionally, in the present analysis we also recovered parallel soft-substrate related paedomorphic simplification in another burrowing aeolidacean family, Embletoniidae (Fig 1), which possesses such definite signs of paedomorphosis as the presence of a small oral veil instead of oral tentacles and only a few cerata. Embletoniidae very well fulfills both the morphological and molecular paedomorphic criteria since it possesses an unequivocal paedomorphic small oral veil, which is commonly assessed within Aeolidacea as an exclusively postlarval character [21, 79], and according to the molecular data is sister to the complex aeolidacean of the family Unidentiidae [19; present study]. Additionally, the small size of both Pseudovermidae and Embletoniidae (common length 2–7 mm) well agrees with the previous conclusions on the paedomorphic origin of meiofauna [6]. A species of the family Embletoniidae was reported to attain a larger size [82] while still maintaining a strongly juvenile external appearance, which further represents the evolutionary processes of deceleration [58] within that basically paedomorphic lineage. Thus, by the presentation here of three independent cases from unrelated aeolidacean families, Xenocratenidae fam.n., Cumanotidae and Embletoniidae, we confirm that soft-substrate burrowing drives paedomorphosis in nudibranchs. The reduction of ceratal rows also occurs in other clades of Aeolidacea, notably in the families Trinchesiidae and Eubranchidae (Fig 2), but is not necessarily linked to the soft-substrate environment. However, here we show that the commonly burrowing *Xenocratena* with only few anterior ceratal rows (3–4) which corresponds to the late postlarval juvenile stage of any complex large Aeolidacea (including the complex, big, non-burrowing Murmaniidae (Figs 1–4), sister to the *Xenocratena*) fulfills the criteria of partial paedomorphic organisation. Instead, the family Trinchesiidae, for example, with its reduced rows of cerata does not demonstrate an adjacent sister relationship to complex ancestors, and although it also shows potential paedomorphic features, they are not easy to immediately link with complex ancestors, in strong contrast with the Murmaniidae–Xenocratenidae case.

Furthermore, the presence of a small oral veil within Aeolidacea is a strong paedomorphic feature and is so far known only within three families; two of them are almost exclusively linked with soft-bottom environments (Pseudovermidae, Embletoniidae) and the third one to brackish waters (within family Trinchesiidae, see [44, 79]), also a substantial driver for the formation of strong paedomorphic organisation. Notably, facultatively burrowing species of the complex family Cumanotidae were shown in the present study as sister to the strongly paedomorphic interstitial Pseudovermidae (Figs 1 and 2) the only representative within all Aeolidacea that displays, besides oral tentacles, also a massive veil-like enlargement of the head (Fig 3). Such a structure can also be considered as a first step to secondarily regaining a postlarval oral veil, also in connection with soft-substrate burrowing habitats.

Several potential mechanisms can explain why a soft-substrate environment is a strong driver of the paedomorphic processes: 1) Small size facilitates movement within sand particles; 2) Reduction of “superfluous” traits and protruding features (like numerous dorsal cerata or other appendages) in paedomorphic animals may enable quick burrowing in soft substrates; 3) Infaunal life style, per se, especially in combination with smaller size can serve as protection against predators, so an organism does not need to maintain more complex protection, as in normal environments. For instance, shells can be weaker or disintegrated into spicules, whereas cerata (a primary means of defense in aeolidacean nudibranchs) would no longer be necessary for protection, and therefore organisms would not need to spend additional energy to maintain such protections, and would reduce them via paedomorphic processes; 4) Commonly smaller paedomorphic animals can open a way to new trophic and niche adaptations by utilizing new food resources within soft substrates, which are also smaller, and correspondingly smaller size can be attained through the process of paedomorphosis.

The presence of more soft-substrate associated nudibranch taxa from different phylogenetically distant families, e.g. *Cerberilla* (Aeolidiidae) and *Paracoryphella* (Paracoryphellidae) (Figs 1 and 2) which are externally similar to Xenocratenidae fam.n. (demonstrating the onset of paedomorphic evolution), and are also within the taxa of the paedomorphic dorid family, Corambidae [34, 38], further considerably strengthens that conclusion. All this evidence shows that soft-substrate infaunal habitats favour paedomorphic evolution. Therefore, we conclude that the ecological criterion can be applied to other soft-substrate burrowing metazoan organisms as additional evidence for paedomorphic origin. Indeed, paedomorphosis as a widespread evolutionary process can occur for various reasons in different habitats and isn’t only restricted to taxa living in a particular environment, but some environments (i.e. soft-substrate marine) more commonly facilitate paedomorphic evolution than others.

### Establishment of a new family Xenocratenidae fam. n

Apart from paedomorphic features, *Xenocratena* acquired several novelties, including highly unusual radular teeth (Figs 2B and 3C), which are different from both its sister family Murmaniidae (Fig 3E) and the distantly related Cumanotidae (Fig 2G), and rather somewhat similar to the otherwise morphologically very different and distantly related (according to the molecular phylogeny) family Aeolidiidae. To accommodate these molecular and morphological disparities within the previously established broad scope of the aeolidacean nudibranch phylogenetic framework [19], here we propose the new family Xenocratenidae fam. n.

- Phylum Mollusca Linne, 1758
- Class Gastropoda Cuvier, 1795
- Order Nudibranchia Cuvier, 1817

### Family Xenocratenidae fam. n

Urn:lsid:zoobank.org:act: 8F5E1024-28C4-4627-8AAE-DB1A6814A53B.

Etymology. After a single included genus and species *Xenocratena suecica* Odhner, 1940.

Diagnosis. Body wide. Notal edge absent. Cerata not stalked, in few continuous rows, no more than five anterior rows (fifth row rudimentary, if present). Rhinophores smooth. Anus acleioproctic. No distinct oral glands. Radula formula 0.1.0. Rachidian radular teeth pectinate, with strong cusp and additional denticles forming a peculiar feather-like structure. Single distal receptaculum seminis present. Vas deferens long, with narrow tubular prostate. Supplementary gland present, inserts to elongated, conical, unarmed copulative organ.

Genera included. *Xenocratena* Odhner, 1940

## Discussion

### Paedomorphic nudibranch *Xenocratena* inhabits the same environment with enigmatic *Xenoturbella*

In the present study, an evolutionary model implying a secondary origin from complex aeolidacean nudibranch ancestors of the multiple paedomorphic nudibranch families Pseudovermidae, Embletoniidae and Xenocratenidae fam.n. is confirmed with novel data. Particularly, the morphological and molecular criteria for the paedomorphic origin of these three nudibranch lineages are fulfilled. Furthermore, previous proposals [6] that soft-substrate infaunal habitats strongly favour the appearance of paedomorphic organisation with several independent cases were supported here using broad-scope molecular phylogeny of a large nudibranch group, Aeolidacea (Fig 1). Therefore, the ecological criteria for paedomorphosis (soft-substrate burrowing) can be applied to other marine invertebrates. For example, the enigmatic vermiform metazoan *Xenoturbella* inhabits fundamentally the same soft-substrata environment with Pseudovermidae, and it shares the same geographic location, fine sand-to mud environment and depth, with *Xenocratena* since the depth distribution of both *Xenoturbella* and *Xenocratena* in the Gullmar fjord overlaps at around 30–40 m [23,24, present study] (Fig 2M). *Xenoturbella bocki* (10–30 mm) is similar in size to *Xenocratena suecica* (10–12 mm) and both are able to burrow in soft substrata. Pseudovermidae and Embletoniidae commonly reach 6 mm, and together with *Xenocratena* and *Xenoturbella*, occupy an intermediate niche between true meiobenthic forms (less than 1 mm) and burrowing macrofauna. It is therefore plausible to suggest that the evolution of the xenacoelomorph *Xenoturbella* might be affected by the same factors that imply evolution from more complex to simplified life forms as evidently shown here for the sister pairs of Cumanotidae–Pseudovermidae (Fig 1), Unidentiidae–Embletoniidae (Fig 1) and Murmaniidae–Xenocratenidae fam. n. (Figs 1 and 3). *Xenoturbella* include shallow and deep-sea clades, which fundamentally have a similar simple body plan and both are able to burrow in soft substrata [8, 10].

According to the present infaunal burrow-driven paedomorphic model of evolution (Fig 4), animals that shifted from just bottom crawling to partly infaunal burrowing habitats as seen in *Xenocratena* (Fig 2C) and *Cumanotus* (Fig 2I) might have been followed by an onset of paedomorphic-driven simplification that “ended up” in a paedomorphic and very simplified *Pseudovermis* (Fig 2J). Pseudovermidae retain such a central molluscan feature as a radula (Fig 2K), however another key molluscan feature, the foot, is already reduced (Fig 2J). This implies that groups which went through strong paedomorphic events may lose many characters that specify their placement in a separate phylum. Another notable example of vermiform molluscs is the clade Aplacophora, where many typical molluscan features have also been lost, but it is currently widely considered as a secondary simplified group [77]. Soft-bottom infaunal habitats may have even led to a loss of almost all molluscan features in the gastropod Rhodopidae, including shell, gills, foot and radula, making this mollusc strongly vermiform not only externally, but also internally [83]. Therefore, in evaluations of potential secondarily reduced modifications (including paedomorphosis) in a metazoan group, it is important to integrate morphological, molecular and ecological criteria, as outlined here.

### Applications of the model of burrow-driven paedomorphosis in nudibranchs to other marine invertebrates, including *Xenoturbella*

Present data thus support that soft-bottom infaunal and burrowing-related habitats strongly favour evolution of simplified body structure and it is not parsimonious to assume that in the Precambrian and early Cambrian eras this driving force of simplification was any different. Several microscopic bilaterian lineages might have emerged, therefore, as the result of a very ancient transition of more complex ancestors to meiobenthic habitats instead of being primarily interstitial [7, 84]. Recent discovery of an extremely small (0.6 mm in length) meiobenthic enteropneust, which has evident paedomorphic features and at the same time is phylogenetically nested within larger macrofaunal hemichordates [85] corroborates the general trend for simplification of infaunal organisms. Applying this evolutionary model of paedomorphic burrowing-related simplification in nudibranch molluscs which inhabit fundamentally the same niche with *Xenoturbella* [8–12] (Fig 2M) it is plausible to suggest that *Xenoturbella* might be paedomorphic and more or less simplified from a more complex ancestor. However, this meiobenthic hemichordate still has evident deuterostomian morphological features, whereas *Xenoturbella* has only a few potential deuterostomian features such as the epithelium structure, which can also be considered a bilaterian plesiomorphy [86].

The simple adult organisation and larva of *Xenoturbella* [87] could be explained by a scenario in which the phylogenetic lineage represented by *Xenoturbella* branched off close to the base of the Deuterostomia [8,11], and hence the ancestral form would be less complex than Ambulacraria and Enteropneusta, but more complex than the modern Xenacoelomorpha. This would explain the persistent uncertainties of the placement of *Xenoturbella* in phylogenomic analyses [88], in which Xenacoelomorpha is either sister to Deuterostomia [9] or sister to deuterostomians and traditional protostomians [10,11], or even in between protostomians and deuterostomians in some trees with less support [10], but in all cases still at the base of deuterostomian and protostomian radiation [9–11]. It was previously proposed that *Xenoturbella* had no evident features of simplification [86], however a recent study shows that anthozoan prebilaterian Cnidaria, which are more complex in several features of morphological organisation (e.g. presence of numerous tentacles, gastric mesenteries) than the vermiform simple gut *Xenoturbella*, have considerably more homeodomain proteins (134 vs. 80) than Xenoacoelomorpha and at the same time Xenoacoelomorpha already possesses all 11 bilaterian homeodomain classes [89]. Notably, while this study had already been completed, a new bilaterian-wide analysis of a 1,173 gene dataset was published and reconfirmed the relationship of Xenoacoelomorpha to Deuterostomia (Ambulacraria) and not as a basal off-shoot of Bilateria [90]. Furthermore, a recent investigation of sperm morphology of *Xenoturbella* shows close similarities with the sperm of hemichordates [91] and the excretory system of Xenoacoelomorpha (despite the absence of specialized nephridia) shows an active transport mechanism similar to Bilateria and not to Cnidaria [92]. Therefore, this profound duality of the phylogenetic position of Xenoacoelomorpha and homedomain organisation coupled with the model that a soft-substrate environment facilitates paedomorphic evolution (Fig 1) suggest that Xenoacoelomorpha originated from an ancestor that was more complex than modern *Xenoturbella*, and that this could fill the gap between cnidarian (as unambiguous sister group to bilaterians) [8–11, 89] and bilaterian radiations. Thus, though a counterargument for an originally simple organisation of the xenacoelomorph is still possible to apply [10,11], the multisource data available for *Xenoturbella* reviewed here fulfill the three proposed criteria for the paedomorphic origin of a metazoan group: 1) *Xenoturbella* possesses specific morphological features (sperm morphology; potentially fine details of epithelia) that are present in a particular taxonomic group (Deuterostomia) but not in other bilaterians and at the same time shows strong paedomorphic external morphology which correspond to the “planula-like” early developmental stages, widely present in Cnidaria and occurring also in some Bilateria; 2) Several molecular analyses (including a recent one) have placed Xenacoelomorpha and *Xenoturbella* within the Deuterostomia instead of being a basal bilaterian offshoot; 3) *Xenoturbella* burrows in soft substrata, a habitat previously proposed and confirmed here as strongly favouring paedomorphic evolution. Presence of larger-sized forms within deep-sea *Xenoturbella* [10] can be explained by subsequent deceleration (“neotenic”) tendencies (favouring a larger size, [32, 58]) on the basis of the generally paedomorphic ancestral patterns.

Ancestral organisation of the sister to the bilaterians phylum, Cnidaria [89], is currently firmly assessed with sedentary anthozans, and not with free-swimming medusas [93, 94]. Further, recent studies of the expression of homeodomain genes allow a direct link between adult sedentary body plans of sponges and cnidarians [95], therefore if sponges would be linked to bilaterians only through the larval stage [96], such similarities would not occur. According to recent phylogenetic analyses, sedentary sponges, and not planktonic ctenophorans, are the most basal animals [97]. Sponges and cnidarians demonstrate complex organisation in both morphology and molecular properties [89; 98, 99], despite the notion that sedentary organisms originated secondarily [85]. Recent paleontological data showed the potential origin of planktonic Ctenophora from sedentary polyps, and not vice versa [100]. Anthozoan cnidarians possess a biphasic ontogenetic cycle [101] with a sedentary adult polypoid stage and a free-living planula larva. This may imply that the last common bilaterian ancestor inherited that sedentary (at the adult stage) and motile (at the larval stage) ontogenetic cycle [102] from a common ancestor with cnidarians. According to the most recent genomic data, Xenacoelomorpha is related to the deuterostomian clades of hemichordates (enteropneusts and pterobranchs) and echinoderms (Ambulacraria) [90]. Recently the apparent earliest representative of echinoderms (without clear attribution to a recognised group of Echinodermata) was redescribed with a possibly assessed straight gut but at the same time with a terminal attaching stalk (without gut inside) and a general sedentary/semi-sedentary appearance [103]. These sedentary features must be absent if such a form of early echinoderms directly descended from the free-living worm-shaped enteropneusts, as commonly assessed currently. A majority of the confirmed early Cambrian echinoderm representatives are sedentary or semisedentary with U-shaped gut, like modern pterobranchs, but not enteropneusts [104–106]. Recent molecular analyses [107, 108] clearly dismiss previous data [109] that the sedentary phylum Pterobranchia is a part of the clade of the free-living Enteropneusta. If Pterobranchia was really modified secondarily from the enteropneust-like groundplan, then the pterobranchs must be nested inside as an internal subclade of the enteropneusts, but pterobranchs instead are confirmed as merely a sister group to Enteropneusta [108]. There is also a large agreement currently that segmentation was not a property of the bilaterian ancestor, but emerged independently in several bilaterian lineages [110–112], that undermines a scenario of a free-living segmented last common bilaterian ancestor [113].

These are only a few striking examples, from numerous currently available data, that the last common bilaterian ancestor can be reliably assessed as sedentary/semi-sedentary at the adult stage [102, 114] and that such a scenario resolves the apparently large disparity [96] between sedentary adult and motile larval parts of the sponge-grade and bilaterians ontogenetic cycles [115]. Xenacoelomorpha do not demonstrate precise molecular phylogenetic relations either to particular sedentary Pterobranchia or to motile recent Enteropneusta (among enteropneusts, semisedentary tubiculous Cambrian forms are also known [103]), but appear as sister to the common deuterostomian echinoderm/hemichordate clade [90]. Therefore, it is reliable to evaluate *Xenoturbella*-like organisms as paedomorphic descendants of the larval part of the more complex ancestral cycle with sedentary/semi-sedentary adult stages, as is common with enteropneusts, pterobranchs and echinoderms. It was specially highlighted previously that causes of the potential simplification of *Xenoturbella* “remains to be uncovered” [8]. The model presented here provides well-supported evidence that a soft-bottom infaunal habitat is a strong driver of paedomoprhic simplification (Figs 1–4) in several lineages of nudibranch molluscs, and using the same scenario the causes of the simplification of Xenacoelomorpha and *Xenoturbella* can be therefore elucidated. This model is further corroborated by the most recent genome-scale data that questioned the simple microscopic body plan of the ancestral bilaterians [116,117], and also by evidence that a rich meiobenthic fauna existed already in the middle to late Cambrian era, with potential implications for the existence of a more complex animal ancestor than these ancient meiobenthic lineages [118].

## Supporting information

S1 Table Primers and PCR programs used in this study. (DOC)

S2 Table GenBank accession numbers and references for all sequences used in this study. (DOC)
