# Supplementary material for: Multiple paedomorphic lineages of soft-substrate burrowing invertebrates: parallels in the origin of Xenocratena and Xenoturbella
Source: PLoS One. 2020 Jan 15;15(1):e0227173. doi: 10.1371/journal.pone.0227173 (PMC6961895; doi:10.1371/journal.pone.0227173)
Supplement: S1 Table — (DOC) [file pone.0227173.s001.doc]

| Name | 5′→3′ | References | Program |
| --- | --- | --- | --- |
| LCO 1490 | GGTCAACAAATCATAAAGATATTGG | Folmer *et al*., 1994 | 95° C 60 s, 35× (95° C 15 s, 45° C 15 s, 72° C 30s), 72° C 7 min |
| HCO 2198 | TAAACTTCAGGGTGACCAAAAAATCA | Folmer *et al*., 1994 |
| 16S arL | CGCCTGTTTAACAAAAACAT | Palumbi *et al*. 2002 | 95° C 3 min, 40× (94° C 15 s, 52° C 15 s, 72° C 30s), 72° C 7 min |
| 16S R | CCGRTYTGAACTCAGCTCACG | Puslednik & Serb, 2008 |
| H3 AF | ATGGCTCGTACCAAGCAGACGG | Colgan *et al*., 1998 | 95° C 3 min, 40× (94° C 15 s, 50° C 15 s, 72° C 30s), 72° C 7 min |
| H3 AR | ATATCCTTGGGCATGATGGTGAC | Colgan *et al*., 1998 |
| 28S C1 | ACCCGCTGAATTTAAGCAT | Dayrat et al. 2001 | 95° C 3 min, 40× (94° C 15 s, 50° C 15 s, 72° C 30s), 72° C 7 min |
| 28S C2 | TGAACTCTCTCTTCAAAGTTCTTTTC | Lê et al, 1993 |
| 18S 3F | GTTCGATTCCGGAG GGGA | Okuzu et al. 2003 | 94° C 6 min, 40× (94° C 60 s, 50° C 60 s, 72° C 90 s), 72° C 6 min |
| 18S bi | GAGTCTCGTTCGTTATCGGA | Okuzu et al. 2003 |
| 18S 2.0 | ATGGTTGCAAAGCTGAAAC | Giribet et al. 1996 |
| 18S 9R | GATCCTTCCGCAGGTTCACCTAC | Giribet et al. 1996 |

**Table S1. Primers and PCR programs used in this study.**

REFERENCES

Dayrat, B., Tillier, A., Lecointre, G., & Tillier, S. New clades of euthyneuran gastropods (Mollusca) from 28S rRNA sequences. *Mol. Phyl. Evol.* 19, 225–235 (2001).

Colgan, D., Macaranas, J., Cassis, G., & Gray, M.R. Histone H3 and U2 snRNA DNA sequences and arthropod molecular evolution. *Austr. J. Zool*. 4, 419–437 (1998).

Folmer, O., Black, M., Hoeh, W., Lutz, R., & Vrijenhoek, R. DNA primers for amplification of mitochondrial cytochrome c oxidase subunit I from diverse metazoan invertebrates. *Mol. Mar. Biol. Biotech.* 3, 294–299 (1994).

Giribet, G., Carranza, S., Baguna, J., Riutort, M., Ribera, C. 1996. First molecular evidence for the existence of a Tardigrada - Arthropoda clade. *Molecular Biology and Evolution* 13, 76-84.

Le, H.L.V., Lecointre, G., & Perasso, R. A 28S rRNA based phylogeny of the Gnathostomes: First steps in the analysis of conflict and congruence with morphologically based cladograms. *Mol Phyl Evol.* 2,

31–51 (1993).

Okuzu A, Schwabe E, Eernisse D, & Giribet G. 2003. Towards a phylogeny of chitons (Mollusca,

Polyplacophora) based on combined analysis of five molecular loci. *Org. Div. Evol.* 3,

281–302.

Palumbi, S.R., Martin, A.P., Romano, S., McMillan, W.O., Stice, L., & Grabowski G. *The simple fool's guide to PCR*. Honolulu: University of Hawaii, 2002.

Puslednik, L., Serb JM. Molecular phylogenetics of the Pectinidae (Mollusca: Bivalvia) and effect of

increased taxon sampling and outgroup selection on tree topology. *Mol Phyl Evol.* 48, 1178–1188 (2008).
