# Supplementary material for: Multiple paedomorphic lineages of soft-substrate burrowing invertebrates: parallels in the origin of Xenocratena and Xenoturbella
Source: PLoS One. 2020 Jan 15;15(1):e0227173. doi: 10.1371/journal.pone.0227173 (PMC6961895; doi:10.1371/journal.pone.0227173)
Supplement: S2 Table — (DOC) [file pone.0227173.s002.doc]

**Table S2. GenBank accession numbers and references for all sequences used in this study.**

| **Species name** | **Voucher** | **Locality** | **COI** | **16S** | **H3** | **28S** | **18S** |
| --- | --- | --- | --- | --- | --- | --- | --- |
| *Aeolidia papillosa* (Linnaeus, 1761) | ZMMU:Op-559 | Russia | KX758257 | KX758252 | KX758261 | - | - |
| *Amphorina pallida* (Alder & Hancock, 1842) | GNM9094 | Scotland | KY129030 | KY128821 | KY128616 | - | - |
| *Antiopella barbarensis* (J. G. Cooper, 1863) | SRR1950942 | - | KX889737 | MK100975 | - | - | MK088213 |
| *Antiopella fusca* (O'Donoghue, 1924) | - | USA | GQ292048 | - | - | - | GQ326887 |
| *Antiopella longidentata* (Gosliner, 1981) | CASIZ176320 | South Africa | HM162749 | HM162673 | HM162582 | - |  |
| *Apata* cf*. pricei* (MacFarland, 1966) | CAS114776 | USA, California | KY129060 | KY128851 | KY128645 | - | - |
| *Apata pricei komandorica* Korshunova, Martynov, Bakken, Evertsen, Fletcher, Mudianta, Saito, Lundin, Schrödl & Picton, 2017 | ZMMU:Op-533 | Russia | MF523386 | MF523413 | MF523311 | **MN850058** | - |
| *Babakina anadoni* (Ortea, 1979) | MNCN15.05/46704 | Spain | HQ616767 | HQ616730 | HQ616796 | - | - |
| *Babakina festiva* (Roller, 1972) | CASIZ182205 | USA, California | - | HQ616736 | HQ616802 | - | - |
| *Baenopsis baetica* (Garcia-Gomez, 1984) | MNCN15.05/53699 | Spain | HQ616755 | HQ616718 | HQ616784 | - | - |
| *Bohuslania matsmichaeli* Korshunova, Lundin, Malmberg, Picton & Martynov, 2018 | ZMMU:Op-600 | Sweden | MG323542 | MG323548 | MG323563 | MG323554 | - |
| *Bonisa nakaza* Gosliner, 1981 | CASIZ176146 | South Africa | HM162746 | HM162670 | HM162579 | - | - |
| *Calmella cavolini* (Vérany, 1846) * | ZMMU:Op-485 | France | MF523319 | MF523448 | MF523245 | MF523515 |  |
| *Calmella cavolini* (Vérany, 1846) * | - | - | - | - | - | - | GU227361 |
| *Catriona aurantia* (Alder & Hancock, 1842) | ZMMU:Op-545 | Norway | KY985467 | MF523458 | MG386404 | MF523524 | - |
| *Catriona columbiana* (O'Donoghue, 1922) | SRR1950949 | - | KX889724 | MK100946 | - | - | MK088189 |
| *Cerberilla mosslandica* McDonald & Nybakken, 1975 | ZMMU:Op-661 | USA | **MN850075** | **MN850067** | **MN850051** | - | - |
| *Chlamylla intermedia* (Bergh, 1899) | ZMMU:Op-480 | Russia | MF523327 | MF523450 | MF523250 | MF523518 | - |
| *Coryphella verrucosa* (Sars M., 1829) * | ZMMU:Op-521 | Russia | MF523375 | MF523421 | MF523300 | MF523494 | - |
| *Coryphella verrucosa* (Sars M., 1829) * | - | USA | - | - | - | - | AF249198 |
| *Cumanotus beaumonti* (Eliot, 1906) | ZMMU:Op-663 | Norway | **MN850076** | **MN850068** | **MN850052** | - | - |
| *Cumanotus beaumonti* (Eliot, 1906) | GNM Gastr 8925 | Sweden | BOLD:AAZ1060 | **-** | **-** | - | - |
| *Cuthona nana* (Alder & Hancock, 1842) | ZMMU:Op-522 | Russia | MF523376 | MF523397 | MF523301 | MF523473 | - |
| *Cuthonella concinna* (Alder & Hancock, 1843) * | ZMMU:Op-523 | Russia | MF523377 | MF523459 | MF523302 | MF523525 | - |
| *Cuthonella concinna* (Alder & Hancock, 1843) * | - | USA, Washington | - | - | - | - | GQ326893** |
| *Cuthonella soboli* Martynov, 1992 | ZMMU:Op-524 | Russia | MF523378 | MF523457 | MF523303 | MF523523 | - |
| *Dendronotus dalli* Bergh, 1879 * | ZMMU:Op-295 | Russia | KM397001 | KM397083 | KM397094 | KM397042 | - |
| *Dendronotus dalli* Bergh, 1879 * | - | - | - | - | - | - | AY165757 |
| *Dendronotus lacteus* (W. Thompson, 1840) | ZMMU:Op-286 | Russia | KC660034 | KC611290 | KC660050 | KC660018 | - |
| *Dendronotus robustus* A. E. Verrill, 1870 | ZMMU:Op-391 | Russia | KM396970 | KM397053 | KM397120 | KM397011 | - |
| *Diaphoreolis viridis* (Forbes, 1840) | ZMMU:Op-537 | Russia | MG266028 | MG266026 | MG266029 | MG266027 | - |
| *Embletonia pulchra* (Alder & Hancock, 1844) | ZFMK Waegele 1118 (Doro) | Croatia | MK091271 | MK100955 | - | - | MK088201 |
| *Embletonia pulchra* (Alder & Hancock, 1844) | ZFMK Em_pulchra_118 | Croatia | MK091272 | MK100956 | - | - | MK088202 |
| *Embletonia* sp. |  | France | MK091273 | MK100957 | - | - | MK088203 |
| *Eubranchus odhneri* (Derjugin & Gurjanova, 1926) | ZMMU:Op-484 | Russia | MF523318 | MF523396 | MF523244 | MF523472 | - |
| *Eubranchus rupium* (Møller, 1842) | - | Helgoland | AF249792 | AF249246 | - | - | AJ224787 |
| *Eubranchus rustyus* (Er. Marcus, 1961)* | CPIC 00611 | **-** | KP871641 | KP871689 | KP871665 | **-** | **-** |
| *Eubranchus rustyus* (Er. Marcus, 1961)* | **-** | USA, Washington | **-** | **-** | **-** | **-** | GQ326905 |
| *Eubranchus tricolor* Forbes, 1838 | ZMMU:Op-525 | Norway | MF523379 | MF523399 | MF523304 | MF523475 | - |
| *Facelina bostoniensis* (Couthouy, 1838) | CAS184184 | USA, New Hampshire | KY129046 | KY128837 | KY128632 | - | - |
| *Favorinus branchialis* (Rathke, 1806) | MNCN15.05/53695 | Spain | HQ616761 | HQ616724 | HQ616790 | - | - |
| *Favorinus elenalexiae* Garcia F. & Troncoso, 2001 | CASIZ178875 | Costa Rica | HM162755 | HM162679 | HM162588 | - | - |
| *Fiona pinnata* (Eschscholtz, 1831) | CASIZ 088586 | USA | KU757491 | KU757615 | KU757600 | KU757652 | KU757607 |
| *Flabellina affinis* (Gmelin, 1791) * | MNCN15.05/53696 | Spain | HQ616753 | HQ616716 | HQ616782 | - |  |
| *Flabellina affinis* (Gmelin, 1791) * | **-** | Spain | - | - | - | - | AY165767 |
| *Flabellinopsis iodinea* (Cooper, 1863) | CAS181313a | USA, California | KY129056 | KY128847 | KY128641 | - | - |
| *Himatina trophina* (Bergh, 1890) * | ZMMU:Op-532 | Russia | MF523389 | MF523460 | MF523314 | MF523526 |  |
| *Himatina trophina* (Bergh, 1890) * | **-** | USA, Washington | - | **-** | **-** | **-** | GQ326910 |
| *Luisella babai* (Schmekel, 1972) * | MNCN15.05/53698 | Spain | HQ616754 | HQ616717 | HQ616783 | - | - |
| *Luisella babai* (Schmekel, 1972) * | - | Spain | - | - | - | AY427449 | AY165768 |
| *Murmania antiqua* Martynov, 2006 | ZMMU:Op-399 | Russia | MF523390 | MF523394 | MF523315 | MF523470 | - |
| *Pacifia amica* Korshunova, Martynov, Bakken, Evertsen, Fletcher, Mudianta, Saito, Lundin, Schrödl & Picton, 2017 | ZMMU:Op-614 | Washington | MG452633 | - | MG452602 | - | - |
| *Pacifia goddardi* Gosliner, 2010 | CAS182590 | California | KY129063 | KY128854 | KY128648 | - | - |
| *Paracoryphella islandica* (Odhner, 1937) | ZMMU:Op-534 | Russia | MF523391 | MF523398 | MF523316 | MF523474 | - |
| *Paraflabellina ischitana* (Hirano & T. E. Thompson, 1990)* | ZFMK Wägele 83 | Italy | - | - | - | - | MK088221 |
| *Paraflabellina ischitana* (Hirano & T. E. Thompson, 1990)* | MNCN15.05/53697 | Spain | HQ616757 | HQ616720 | HQ616786 | - | - |
| *Phestilla lugubris* (Bergh, 1870)* | ZFMK Wägele 501 | Indonesia | MK091284 | MK100983 | - | - | MK088222 |
| *Phestilla lugubris* (Bergh, 1870)* | CAS177437 | Philippines | - | - | KY128660 | - |  |
| *Phestilla melanobrachia* Bergh, 1874 | ZFMK Wägele 518 | Indonesia | MK091287 | MK100985 | - | - | MK088223 |
| *Pseudovermis paradoxus* Perejaslavtseva, 1891 | ZMMU:Op-178-1 | Russia | MK059477 | **MN850069** | **MN850053** | **MN850059** | **-** |
| *Pseudovermis paradoxus* Perejaslavtseva, 1891 | ZMMU:Op-178-2 | Russia | **MN850077** | **MN850070** | **MN850054** | **MN850060** | **-** |
| *Sakuraeolis enosimensis* (Baba, 1930) | CASIZ178876 | USA, California | HM162758 | HM162682 | HM162591 | - | - |
| *Samla bicolor* (Kelaart, 1858) | ZMMU:Op-68 | Vietnam | MF523383 | MF523436 | MF523308 | MF523503 | - |
| *Tergipes tergipes* (Forsskål in Niebuhr, 1775) * | ZMMU:Op-662 | Barents Sea | **MN850078** | **MN850071** | - | **MN850061** | - |
| *Tergipes tergipes* (Forsskål in Niebuhr, 1775) * | - | - | - | - | - | - | AF249197 |
| *Trinchesia caerulea* (Montagu, 1804) * | ZMMU:Op-622 | Norway | MG266024 | MG266022 | MG266025 | MG266023 | - |
| *Trinchesia caerulea* (Montagu, 1804) * | - | North Sea | - | - | - | - | AF249199 |
| *Tritonia nilsodhneri* Marcus Ev., 1983 * | CASIZ176219 | South Africa | HM162716 | HM162641 | HM162548 | - | - |
| *Tritonia nilsodhneri* Marcus Ev., 1983 * | - | Spain | - | - | - | - | AF249200 |
| *Tritonia plebeia* Johnston, 1828 | ZMMU:Op-572 | Norway | KX788134 | KX788122 | - | KX788132 | - |
| *Unidentia aliciae* Korshunova, Mehrotra, Arnold, Lundin, Picton & Martynov, 2019 | ZMMU Op-634 | Thailand | MK204486 | MK204483 | MK204489 | - | - |
| *Unidentia nihonrossija* Korshunova, Martynov, Bakken, Evertsen, Fletcher, Mudianta, Saito, Lundin, Schrödl & Picton, 2017 | ZMMU:Op-517 | Japan | MF523385 | MF523464 | MF523310 | - | - |
| *Unidentia sandramillenae* Korshunova, Martynov, Bakken, Evertsen, Fletcher, Mudianta, Saito, Lundin, Schrödl & Picton, 2017 | ZMMU:Op-617 | Indonesia | MG452632 | MG452683 | MG452601 | - | - |
| *Unidentia* sp. Millen & Hermosillo, 2012 | SRR3726696 | Australia | KX889750 | MK100996 | - | - | MK088235 |
| *Xenocratena suecica* Odhner, 1940 | GNM Gastr 9816 | Sweden | **MN850079** | **MN850072** | **MN850055** | **MN850062** | **-** |
| *Xenocratena suecica* Odhner, 1940 | GNM Gastr 9770-1 | Norway | **MN850080** | **MN850073** | **MN850056** | **MN850063** | **MN850065** |
| *Xenocratena suecica* Odhner, 1940 | GNM Gastr 9770-2 | Norway | **MN850081** | **MN850074** | **MN850057** | **MN850064** | **MN850066** |
| *Zelentia ninel* Korshunova, Martynov & Picton, 2017 | ZMMU:Op-509 | Russia | KY952178 | MF523400 | MF523242 | MF523476 | - |
| *Zeusia hyperborea* Korshunova, Zimina & Martynov, 2017 | ZMMU:Op-557 | Russia | KX758256 | KX758251 | KX758260 | - | - |

* data for two specimens of the same species were concatenated.

** *Cuthonella concinna* was improperly identified as *Cuthona cocoachroma* in Shields, 2009

REFERENCES

Bleidissel S. and Preisfeld A. Direct submission to GenBank.

Carmona, L, Gosliner, T.M., Pola, M., Cervera, J.L. A molecular approach to the phylogenetic status of the aeolid genus *Babakina* Roller, 1973 (Nudibranchia). *J. Moll. Stud.* 77, 417–422 (2011).

Cella, K., Carmona, L, Ekimova, I., Chichvarkhin, A., Schepetov, D., Gosliner, T.M. A Radical

Solution: The phylogeny of the nudibranch family Fionidae. *PLoS ONE* 11, e0167800 (2016).

Goodheart, J. A.; Bleidißel, S.; Schillo, D.; Strong, E. E.; Ayres, D. L.; Preisfeld, A.; Collins, A. G.; Cummings, M. P.; Wägele, H. Comparative morphology and evolution of the cnidosac in Cladobranchia (Gastropoda: Heterobranchia: Nudibranchia). Frontiers in Zoology. 15: 43 (2018).

Ekimova, I., Korshunova, T., Schepetov, D., Neretina, T., Sanamyan, N., Martynov, A. Integrative systematics of northern and Arctic nudibranchs of the genus *Dendronotus* (Mollusca, Gastropoda), with descriptions of three new species. *Zool.l J. Linn. Soc.* 173, 841–886 (2015).

Flammensbeck C.K., Haszprunar G., Korshunova T. Martynov A.V., Neusser T.P., Jörger K.M. *Pseudovermis paradoxus* 2.0—3D microanatomy and ultrastructure of a vermiform, meiofaunal nudibranch (Gastropoda, Heterobranchia). Org. Divers. Evol. 1-22 (2019)

Korshunova, T.. Lundin, K., Malmberg, K.. Picton, B., Martynov, A. First true brackish-water nudibranch mollusc provides new insights for phylogeny and biogeography and reveals paedomorphosis-driven evolution. PLOS ONE. 13, e0192177 (2018).

Korshunova, T., Martynov, A., Bakken, T., Evertsen, J., Fletcher, K., Mudianta, W., Saito, H., Lundin, K., Schrödl, M., Picton, B. Polyphyly of the traditional family Flabellinidae affects a major group of Nudibranchia: aeolidacean taxonomic reassessment with descriptions of several new families, genera, and species (Mollusca, Gastropoda). ZooKeys 717, 1–139 (2017).

Korshunova, T., Martynov, A., Picton B. Ontogeny as an important part of integrative taxonomy in tergipedid aeolidaceans (Gastropoda: Nudibranchia) with a description of a new genus and species from the Barents Sea. *Zootaxa* 4324 1, 1–22 (2017).

Korshunova T., Mehrotra R., Arnold S., Lundin K., Picton B., Martynov A. The formerly enigmatic Unidentiidae in the limelight again: a new species of the genus Unidentia from Thailand (Gastropoda: Nudibranchia). Zootaxa. 4551 (5), 556–570 (2019).

Korshunova, T., Sanamyan, N., Zimina, O., Fletcher, K., & Martynov, A. Two new species and a remarkable record of the genus *Dendronotus* from the North Pacific and Arctic oceans (Nudibranchia). *ZooKeys* 630, 19–42 (2016).

Korshunova T., Zimina O. & Martynov A. (2017). Unique pleuroproctic taxa of the nudibranch family Aeolidiidae from the Atlantic and Pacific Oceans, with description of a new genus and species. J. Moll. Stud. 83, 409–421.

Mahguib, J., Valdés, Á. Molecular investigation of the phylogenetic position of the polar nudibranch *Doridoxa* (Mollusca, Gastropoda, Heterobranchia). *Polar Biol.* 38, 1369 (2015).

Pola, M., Gosliner T. The first molecular phylogeny of cladobranchian opisthobranchs (Mollusca, Gastropoda, Nudibranchia). (2010). *Molecular phylogenetics and evolution* 56 3: 931-41.

Shields, C. *Nudibranchs of the Ross Sea, Antarctica: phylogeny, diversity, and divergence*. Ph.D. thesis. 637 (2009).

Trickey, J. Phylogeography and molecular systematics of the rafting aeolid nudibranch *Fiona pinnata* (Eschscholtz, 1831). Ph.D. [Thesis](https://ourarchive.otago.ac.nz/browse?type=type&value=Thesis). University of Otago (2013).

Vonnemann, V., Schrödl, M., Klussmann-Kolb, A., & Wägele, H. Reconstruction of the phylogeny of the Opisthobranchia (Mollusca: Gastropoda) by means of 18s and 28s rRNA gene sequences. *J. Moll. Stud*. 71, 113–125 (2005).

Wagele, H., Vonnemann, V., & Wagele, W. Toward a phylogeny of the Opisthobranchia. In Lydeard, C. & Lindberg, D.R. (Eds.), Molecular systematics and phylogeography of mollusks. (pp. 185-228). Washington, DC: Smithsonian (2003).

Wollscheid-Lengeling E, Boore J, Brown W, & Wägele H. The phylogeny of Nudibranchia (Opisthobranchia, Gastropoda, Mollusca) reconstructed by three molecular markers. *Org. Div. Evol.* 1, 241–256 (2001).
